# Adaptive Logit Fusion for Mitigating Class Imbalance in Multi-Category Sperm Morphology Assessment

**DOI:** 10.3390/life16030438

**Published:** 2026-03-09

**Authors:** Emin Can Özge, Hamza Osman Ilhan, Gorkem Serbes, Hakkı Uzun, Ali Can Karaca, Merve Huner Yigit

**Affiliations:** 1 Research and Development, Siemens A.S., Istanbul 34870, Türkiye; emincan.ozge@siemens.com; 2 Department of Computer Engineering, Yildiz Technical University, Istanbul 34220, Türkiye; hoilhan@yildiz.edu.tr (H.O.I.); ackaraca@yildiz.edu.tr (A.C.K.); 3 Department of Biomedical Engineering, Yildiz Technical University, Istanbul 34220, Türkiye; gserbes@yildiz.edu.tr; 4 Department of Urology, Faculty of Medicine, Recep Tayyip Erdoğan University, Rize 53000, Türkiye; hakki.uzun@erdogan.edu.tr; 5 Department of Biochemistry, Faculty of Medicine, Recep Tayyip Erdoğan University, Rize 53000, Türkiye

**Keywords:** sperm morphology, adaptive logit fusion, ensemble learning, ResNet50V2, EfficientNetV2-S, infertility

## Abstract

Sperm morphology is one of the most critical indicators of male fertility. This paper presents a deep learning-based approach to classify sperm cells into 18 morphological classes, including one normal and 17 abnormal types. Two state-of-the-art convolutional neural networks, EfficientNetV2-S and ResNet50V2, are employed and fine-tuned using a class-weighted loss function together with extensive data augmentation to improve generalization under class imbalance. Automatic mixed precision training is adopted to reduce memory consumption and accelerate the training process. An ensemble strategy is subsequently constructed by linearly fusing the logits of both architectures, where the fusion weight is optimized to maximize recall, precision, and overall F1-score. Experimental results show that the proposed ensemble achieves an overall accuracy of 70.94%, consistently outperforming the individual models. Sperm cells with pronounced structural abnormalities, such as PinHead and DoubleTail, are classified with high accuracy, whereas less visually distinctive defects result in comparatively lower performance. These findings demonstrate the potential of CNN-based ensemble models to provide consistent and reliable automated sperm morphology classification.

## 1. Introduction

The morphology of human sperm—particularly the size and structure of the head, neck, and tail—is one of the most critical signs of male fertility. Abnormalities in these structures can cause problems with motility, reduced fertilization capability, and infertility. Traditionally, sperm morphology has been assessed manually through microscopic examination, relying on subjective evaluations that are prone to inter-observer variability and require substantial expertise. Some studies have shown that manual sperm morphology assessment suffers from inter- and intra-observer variability, even among experienced embryologists, which makes results difficult to reproduce and reduces diagnostic consistency across laboratories [1,2]. Therefore, there is a strong need for automated, precise, and reproducible methods to accurately identify and classify sperm morphological abnormalities.

Deep convolutional neural networks (CNNs) have demonstrated strong performance in medical image analysis due to their powerful feature extraction capabilities [3]. Recent studies, including those by Yüzkat et al. [4] and Guo et al. [5], have successfully applied CNN-based approaches to the sperm morphology classification task using backbone networks such as ResNet and DenseNet. However, CNN-based workflows encounter several challenges in fine-grained morphology recognition. Models often struggle to distinguish between classes that differ only by slight shape or structural changes, such as ThinNeck versus AsymmetricNeck or TaperedHead versus PyriformHead, where class boundaries are defined by subtle differences [5,6]. In addition, CNN-based models often exhibit limited robustness to changes in image appearance caused by intra-class variations in staining protocols, microscope magnification, illumination conditions, and imaging devices. Such domain shift effects have been shown to degrade model generalization when acquisition conditions vary [7,8,9,10,11]. Furthermore, CNNs tend to prioritize local texture cues over global shape information, making the recognition of subtle structural differences particularly difficult [12,13,14].

Sperm morphology exhibits a wide range of abnormal patterns. For example, sperm heads may appear pyriform, tapered, or amorphous, while neck and tail defects can be multi-segmented, asymmetric, or twisted. In addition, sperm morphology datasets often suffer from severe class imbalance, where rare abnormalities such as DoubleTail or LongTail are substantially underrepresented and may be overshadowed by more frequent morphological patterns during training. Class imbalance is a well-known challenge in medical image classification, as deep learning models tend to favor dominant classes unless specific countermeasures such as loss reweighting or sampling strategies are applied [15,16]. Finally, subtle intra-class variations among visually similar abnormalities (e.g., ThinNeck versus AsymmetricNeck) introduce additional ambiguity, making reliable discrimination particularly challenging for classification models. Such subtle morphological differences require models to capture both global contextual information and localized structural cues, motivating the adoption of attention-based or multi-scale feature learning approaches in visual classification tasks [17,18].

Although previous studies have benchmarked multiple deep learning architectures on the sperm morphology task, most of them rely on single backbone models. While these approaches provide strong feature representations, they may not fully exploit complementary information across different architectural designs. In addition, confusion among visually similar abnormality categories remains a persistent issue, as reported in prior studies.

In this context, Aktas et al. [8] introduced the Hi-LabSpermMorpho dataset, which includes 18 classes of sperm morphology obtained using various staining methods. Their baseline experiments, involving more than 30 different deep learning models, demonstrated that EfficientNetV2 variants achieved the highest classification accuracy; however, confusion among visually similar abnormality categories persisted. These findings suggest that, although single architectures can achieve competitive performance, integrating complementary models may yield additional benefits.

To address these challenges, this study analyzes 18 sperm morphology classes using the BesLab subset of the Hi-LabSpermMorpho dataset under a five-fold cross-validation strategy. In each fold, approximately 20% of the data is reserved for testing, ensuring that every sample is evaluated exactly once throughout the experimental protocol. Independent model instances are trained and evaluated for each fold, and the results are aggregated to provide a robust assessment of generalizability. Two complementary CNN architectures, EfficientNetV2-S and ResNet50V2, are used to capture diverse feature representations. EfficientNetV2-S leverages compound scaling and mobile inverted bottleneck blocks, whereas ResNet50V2 adopts residual skip connections with a pre-activation structure to facilitate stable gradient flow [19].

Automatic mixed precision training is adopted to accelerate computation without compromising model accuracy. A class-weighted loss function is used to mitigate class imbalance, and data augmentation techniques are applied to improve generalization. The final prediction is obtained by combining the two architectures’ logit outputs through a weighted fusion strategy, where the fusion coefficient is optimized via grid search to achieve the best overall performance. Classification performance is evaluated using precision, recall, and F1-score, and the results are summarized with confusion matrices to provide a comprehensive view of the model’s behavior.

The main contributions of this study are summarized as follows:

- An adaptive logit-level fusion framework combining EfficientNetV2-S and ResNet50V2 for 18-class sperm morphology.
- A systematic five-fold cross-validation protocol with independent model training per fold to ensure robust generalization assessment.
- A class-weighted optimization strategy to mitigate severe class imbalance in rare morphological categories.
- A comprehensive experimental evaluation including ablation analysis, statistical significance testing, area under the receiver operating characteristic curve (AUROC) and area under the precision–recall curve (AUPRC) analysis, and computational complexity benchmarking.
- External validation on the HuSHeM (Human Sperm Head Morphology) dataset to assess robustness under dataset shift.

The remainder of this paper is organized as follows: Section 2 describes the dataset characteristics, preprocessing strategy, model architectures, training protocol, and ensemble formulation. Section 3 presents the experimental results. Section 4 discusses the findings in relation to existing studies. Finally, Section 5 concludes the study and outlines future research directions.

## 2. Materials and Methods

**Figure 1:**
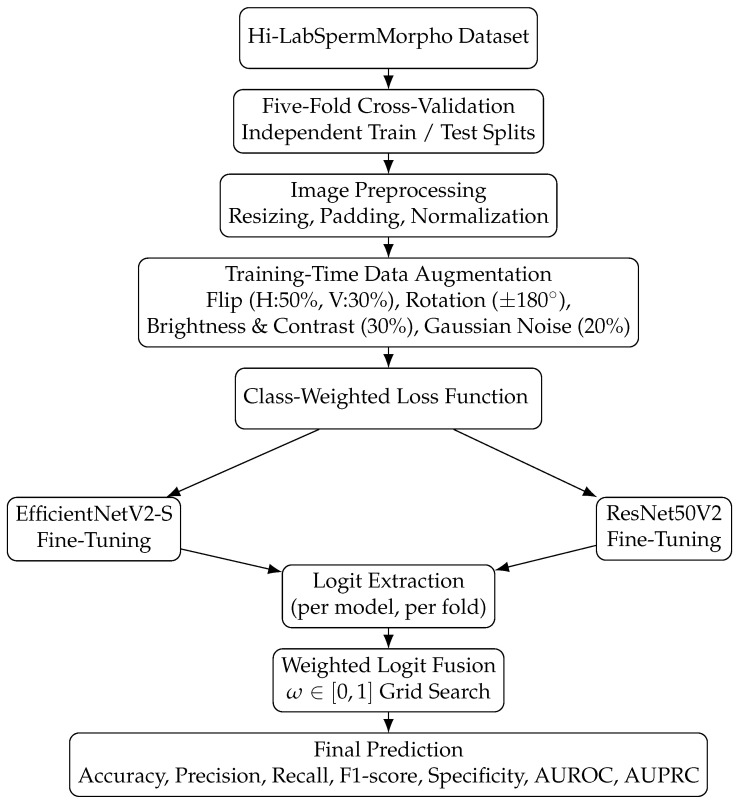
Overview of the proposed framework. Following preprocessing and training-time augmentation, EfficientNetV2-S and ResNet50V2 are independently fine-tuned under five-fold cross-validation using class-weighted loss. Their logits are fused through weighted integration and evaluated using accuracy, precision, recall, F1-score, specificity, AUROC, and AUPRC.

This study investigates 18 sperm morphology classes (17 abnormal and 1 normal) using a five-fold cross-validation design on the Hi-LabSpermMorpho dataset introduced by Aktas et al. [8]. In each fold, the dataset is divided into mutually exclusive training and test subsets, ensuring that no image appears more than once in the test sets of each fold. For every fold, independent model instances are trained and evaluated, rather than reusing a single model iteratively. Performance metrics obtained from the test sets of all five folds are subsequently aggregated to provide a comprehensive and robust assessment of model generalization. An overview of the proposed methodology is illustrated in Figure 1.

### 2.1. Dataset Information

**Figure 2:**
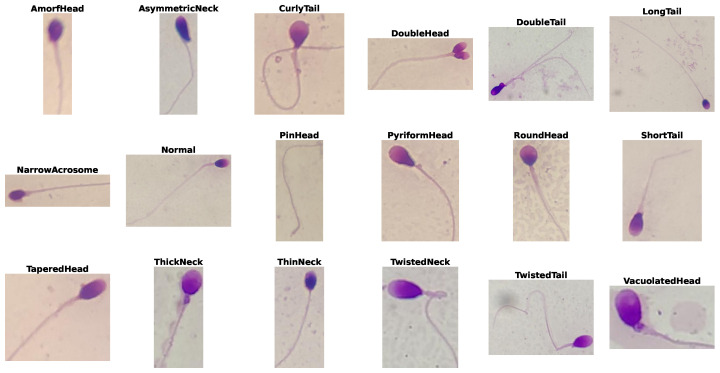
Representative examples of all 18 sperm morphology classes from the BesLab subset of the Hi-LabSpermMorpho dataset. Each image corresponds to a distinct morphology category (one normal and 17 abnormal classes) used for model training and evaluation.

The Hi-LabSpermMorpho dataset was created using a standardized and clinically approved method to guarantee consistency and high-quality morphological annotations. Semen samples were obtained from patients at a clinical andrology laboratory with ethical permission and informed consent, following the guidelines set by the World Health Organization (WHO) for semen analysis. To improve the visibility of detailed morphological structures, three distinct Diff-Quick staining kits (BesLab, HistoPLUS, and GBL) were utilized in the sample preparation process, enabling the assessment of staining-induced visual variations. Following staining, images were captured using bright-field microscopy at a magnification of 100×. Image acquisition was carried out using a specially designed device that connects a regular mobile phone camera to the microscope eyepiece, enabling the capture of high-resolution RGB (Red, Green, Blue) images without requiring specialized laboratory cameras. This low-cost and scalable acquisition system enables wider use in laboratories that have different levels of technical resources. All images were then anonymized and categorized by expert embryologists into 18 morphology classes that include abnormalities of the head, neck, and tail, along with a class for normal specimens [8]. All experiments were conducted exclusively on the BesLab-stained subset of the Hi-LabSpermMorpho dataset to ensure staining consistency and to enable a fair comparison with prior work, following the reference evaluation protocol adopted in the original dataset study. Representative examples of all 18 morphology classes are shown in Figure 2.

Despite this standardized acquisition and annotation process, the images exhibit varying spatial resolutions due to differences in acquisition conditions and cropping during dataset preparation. Since convolutional neural networks require fixed-size inputs, this variability introduces an additional challenge during model training. To address this, a resizing and padding strategy was applied during preprocessing to ensure compatibility with CNN architectures while minimizing distortion and preserving morphological integrity.

### 2.2. Dataset Partitioning and Class Distribution

The dataset includes images from 18 sperm morphology classes, comprising one normal class and 17 abnormal classes representing defects in the head, neck, and tail regions. The head abnormality classes include amorphous, tapered, double head, pyriform, PinHead, vacuolated, NarrowAcrosome, and round head. Neck abnormality classes include a thin neck, a thick neck, a twisted neck, and an asymmetric neck. Tail abnormality classes include a double tail, a curly tail, a long tail, a short tail, and a twisted tail.

A pronounced class imbalance exists across these categories. Several abnormality types are severely underrepresented, particularly long-tail, double-head, and thin-neck defects. To address this issue, a class-weighted loss function is employed during training to reduce the dominance of frequent classes and encourage more balanced learning across all categories. This strategy improves the model’s sensitivity to rare morphological patterns and contributes to a more equitable performance distribution. Similar approaches have been widely adopted in sperm morphology analysis and medical image classification to mitigate the effects of class imbalance, as demonstrated by Shahzad et al. [20] and Hellín et al. [16].

**Figure 3:**
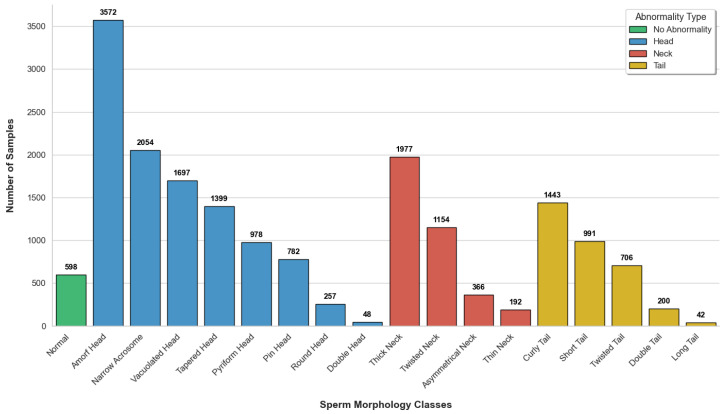
Class distribution of the BesLab subset across all 18 sperm morphology classes. The histogram highlights severe class imbalance, with several rare abnormalities containing substantially fewer samples than dominant categories.

The class distribution of the BesLab subset across the five folds is shown in Figure 3, which reports the number of samples in each morphology category. It should be explicitly noted that no offline resampling or synthetic rebalancing was performed. Class imbalance was handled using a class-weighted cross-entropy loss, which adjusts the optimization without changing sample frequencies. Furthermore, data augmentation was applied at runtime during training and did not modify the underlying class distribution. Therefore, the original class distribution figure still reflects the effective dataset used for training and evaluation. To further ensure reliable evaluation under class imbalance, a five-fold evaluation protocol was employed. In this setup, the dataset was organized into five non-overlapping folds. For each fold, a separate model was trained from scratch using the corresponding training subset and evaluated on its associated test subset. This procedure was repeated independently for all five folds. This design allows performance consistency across different data partitions to be assessed without information leakage.

On average, each fold contains approximately 3691±7 samples. The per-class sample counts exhibit minimal variation across folds, remaining within approximately ±1–2 samples. This near-uniform class distribution across folds supports a fair and stable evaluation despite the pronounced class imbalance present in the dataset.

### 2.3. Image Preprocessing and Augmentation

Dataset partitioning into training and test subsets was performed prior to any preprocessing or augmentation operations to prevent information leakage. By strictly separating the data before any image-level operations, the independence of the test set was preserved, allowing a fair and unbiased evaluation of the model’s generalization performance. To ensure consistent input quality and enhance the robustness of the proposed models, a series of preprocessing and data augmentation steps was applied exclusively to the training sets within each fold. These operations are designed to preserve discriminative morphological features while improving generalization across varying imaging conditions. Data augmentation was not performed on the test sets, and the evaluation was carried out solely on the original, unaugmented images, ensuring a fair and leak-free evaluation.

**Figure 4:**
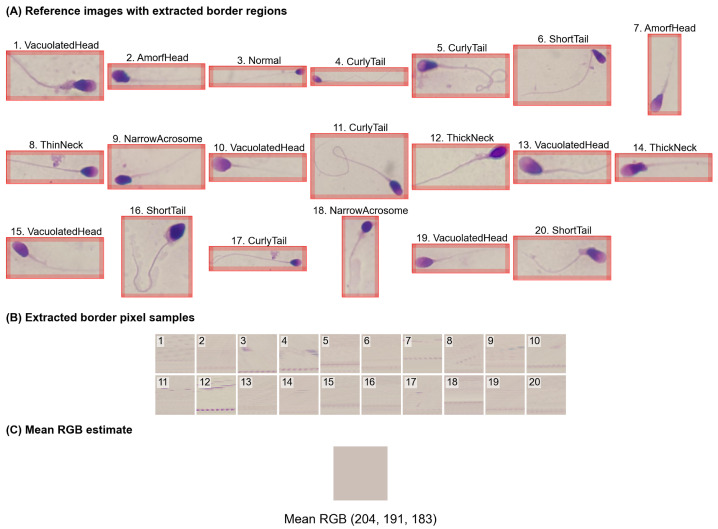
Determination of dataset-specific padding color from background regions. (**A**) Twenty representative sperm images with extracted background border regions highlighted. (**B**) Zoomed-in views of the extracted border pixel samples used for background color estimation. (**C**) Mean RGB color computed over all extracted background pixels, which was used as the fixed padding color during preprocessing.

In the image processing stage, all images were resized to a fixed resolution of 240×240 pixels to address the non-uniform image resolutions in the dataset and to meet the input requirements of the CNN models. To preserve the original aspect ratio and avoid geometric distortion of sperm morphology, padding was applied when necessary. The padding color (RGB: 204, 191, 183) was determined by computing the average color of background border regions extracted from 20 representative sample images, as shown in Figure 4.

In terms of data augmentation, to improve model robustness and reduce overfitting, a comprehensive data augmentation pipeline was applied exclusively during the training phase of each fold. These augmentations aim to increase intra-class variability, simulate realistic acquisition conditions, and enhance the model’s invariance to changes in orientation, illumination, and noise. Given the wide morphological diversity of sperm cells and the presence of class imbalance, augmentation plays a critical role in exposing the network to a broader distribution of plausible variations without altering the underlying class semantics.

Data augmentation was implemented during training using random operations instead of increasing the dataset size by a fixed amount. In each training epoch, every image in the training set was independently transformed according to predefined probabilities. As a result, many training samples were stochastically augmented in each epoch, and the same image was presented in different augmented variants across epochs.

The following augmentation strategies were applied prior to each fold’s training phase:

- Random Geometric Transforms: Horizontal flips (*p* = 0.5), vertical flips (*p* = 0.3), and random rotations within the range of $\left[-180^{\circ },180^{\circ }\right]$ were applied.
- Photometric Adjustments: With a 30% probability, random brightness and contrast adjustments were applied, and with a 20% probability, Gaussian noise was added to simulate sensor-induced noise.
- Normalization: Pixel intensities were normalized using ImageNet statistics (mean = [0.485, 0.456, 0.406], std = [0.229, 0.224, 0.225]).
- Tensor Conversion: Transforming images into tensor representations for neural network input.

**Figure 5:**
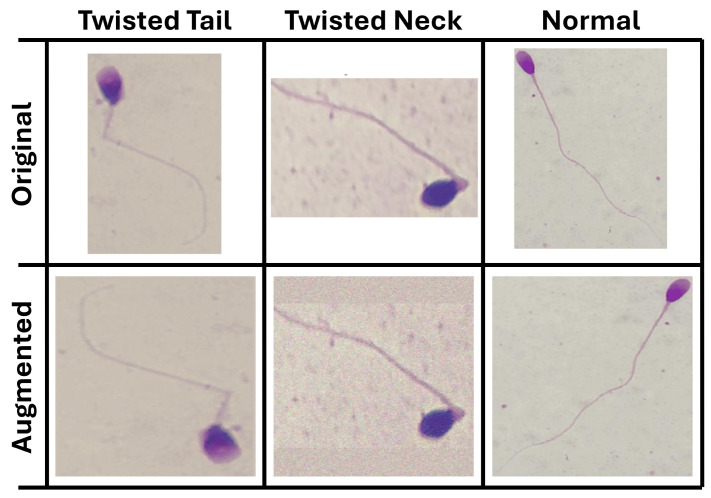
Examples of training-time augmentation applied to three morphology classes (TwistedTail, TwistedNeck, and Normal). Each row shows the original image and representative augmented variants generated using geometric and photometric transformations.

Augmentation was applied only to the training data. The test set was processed using fixed preprocessing steps, including resizing and ImageNet-based normalization without any random transformations. This kept the training and test data separate and prevented data leakage. Figure 5 illustrates examples of the outcomes of the applied data augmentation pipeline for the three original image categories, namely the Twisted Tail, Twisted Neck, and Normal classes, each displayed with their corresponding augmented variants.

### 2.4. Deep Learning Architectures and Hyperparameter Tuning

Two state-of-the-art CNN architectures with distinct design principles were utilized in this study to model complex sperm morphology patterns. Each network was fine-tuned through systematic hyperparameter optimization to achieve stable training and robust performance across all folds.

From a mathematical perspective, both architectures rely on stacked convolutional mappings to extract hierarchical morphological features. A standard convolutional layer can be expressed as

(1) $$y_{i,j,k}=\sum_{c=1}^{C_{\mathrm{in}}}\sum_{u=1}^{K_{h}}\sum_{v=1}^{K_{w}}W_{u,v,c,k}\;x_{i+u-1,\;j+v-1,\;c}+b_{k},$$

where *x* denotes the input feature tensor, *W* represents learnable convolutional kernels, $b_{k}$ is the bias term, and *y* is the resulting activation. Through successive nonlinear transformations, the networks progressively encode structural head, neck, and tail characteristics into high-level feature representations.

Both backbone architectures were trained under identical optimization settings. The initial learning rate was set to $1\times 10^{-4}$ and was reduced by a factor of two when the validation metrics plateaued for three consecutive epochs. Weight decay was set to $1\times 10^{-4}$ to reduce overfitting. Early stopping was applied after 10 non-improving epochs based on validation accuracy. A batch size of 8 was selected based on empirical validation performance. Although GPU (graphics processing unit) memory allowed larger batch sizes (16 and 32), those configurations did not achieve comparable weighted F1-scores and exhibited earlier performance saturation under the same early stopping criteria. Therefore, a batch size of 8 yielded the most stable convergence and the best overall generalization across folds.

Mini-batch training was implemented using the PyTorch (version 2.9.0) DataLoader, with shuffling enabled for training subsets and disabled for validation subsets. No weighted sampling strategy was employed. Class imbalance was addressed exclusively through class-weighted cross-entropy loss.

#### 2.4.1. EfficientNetV2-S

The EfficientNetV2 architecture, specifically the EfficientNetV2-S variant, was adopted due to its strong performance-to-efficiency ratio and its suitability for detailed visual recognition tasks. Prior studies on the Hi-LabSpermMorpho dataset have shown that EfficientNetV2 variants achieve the highest classification accuracy among a wide range of deep learning architectures, demonstrating their effectiveness in capturing fine-grained sperm morphology features [8]. Motivated by this consistent performance, EfficientNetV2-S was selected as one of the backbone models to support the proposed adaptive logit fusion-based ensemble framework. The model was fine-tuned to adapt pretrained representations to sperm morphology characteristics, while carefully selected hyperparameters were used to ensure stable optimization and effective regularization. The main architectural and training details are summarized as follows:

- Pretraining and Feature Extraction: The model was initially trained on a large-scale dataset (ImageNet-21k) and then fine-tuned on a more specific dataset (ImageNet-1k), capturing both generic low-level features and mid-level texture patterns [19]. EfficientNetV2-S employs mobile inverted bottleneck convolution (MBConv) blocks, including early-stage fused MBConv layers, and a compound scaling strategy that uniformly scales network depth, width, and input resolution.
- Model-Specific Setting:
  – Dropout rate of 0.7 for regularization.

#### 2.4.2. ResNet50V2

The ResNet50V2 architecture was selected as a complementary backbone due to its strong representation capacity and proven stability in training deep convolutional networks. Residual network architectures such as ResNet and its variants have been widely and successfully applied to medical image classification, where they consistently demonstrate strong performance in capturing subtle and fine-grained diagnostic patterns [3,8,9]. In particular, the residual learning paradigm and pre-activation design of ResNet50V2 facilitate stable gradient propagation and robust feature learning, making it especially effective for visual classification tasks such as sperm morphology analysis.

Formally, residual learning can be described as follows:

(2) y=F(x,W)+x,

where F(x,W) denotes the residual mapping learned by stacked convolutional layers and x represents the identity shortcut connection. This formulation enables the network to learn refinements over existing morphological representations rather than complete transformations, improving optimization stability in deep architectures.

The key architectural characteristics and training considerations are summarized below.

- Architecture and Feature Learning: ResNet50V2 is a deep residual network with 50 layers that employs stacked residual blocks with identity skip connections and a pre-activation layout. This structure facilitates the training of very deep models by preserving gradient flow, enabling the network to learn complex and deep feature hierarchies that are robust to subtle morphological variations [21].
- Pretraining and Distillation: In this study, a ResNet50V2 model pretrained under the Big Transfer (BiT) framework was used. The selected pretrained checkpoint benefited from knowledge distillation during the pretraining phase on large-scale data, as provided by the original BiT training procedure [22]. In this study, no additional knowledge distillation was applied during training.
- Model-Specific Setting:
  – Dropout rate of 0.5 for moderate regularization.

### 2.5. Mixed Precision Training

Both networks were trained using PyTorch’s automatic mixed precision (AMP) [23,24]. This approach optimizes matrix operations by executing convolutions and matrix multiplications in half precision (FP16) while retaining full precision (FP32) for numerically sensitive layers such as reductions, softmax, and batch normalization [23,24]. AMP’s autocasting mechanism automatically selects the appropriate precision for each operation, ensuring that the model’s convergence and final accuracy remain on par with full FP32 training [23]. AMP also reduces GPU memory consumption by almost halving the memory footprint of activations and speeds up training [25]. Apart from AMP, PyTorch’s GradScaler was utilized to dynamically scale the loss, preventing gradient underflow that may occur in FP16 [23]. This scaling maintains gradients within a numerically representable range, ensuring stable convergence [24].

### 2.6. Ensemble Strategy

To combine the complementary representations of the two backbones, fusion was performed in logit space. Unlike probability averaging, this approach preserves linear separability before softmax normalization and reduces distortions caused by probability compression. Each backbone architecture was trained independently under identical cross-validation splits. Logit fusion was applied only during inference; no joint training was performed.

Let $z^{\left(E\right)}\in R^{C}$ and $z^{\left(R\right)}\in R^{C}$ denote the logit vectors produced by EfficientNetV2-S and ResNet50V2, respectively, where C=18 represents the number of morphology classes. The fused logits are defined as a convex combination as follows:

(3) $$z^{\left(\mathrm{ens}\right)}=\left(1-\omega \right)z^{\left(E\right)}+\omega z^{\left(R\right)},\;\omega \in \left[0,1\right]$$

The predicted class label is obtained directly from the fused logits as follows:

(4) $$\hat{y}=\arg\max_{c}z_{c}^{\left(\mathrm{ens}\right)}$$

Since the softmax function is strictly monotonic, applying arg max after softmax yields identical class assignments. For probability-based evaluation metrics such as AUROC and AUPRC, the class probabilities are computed as follows:

(5) $$p_{c}=\frac{\exp\left(z_{c}^{\left(\mathrm{ens}\right)}\right)}{\sum_{j=1}^{C}\exp\left(z_{j}^{\left(\mathrm{ens}\right)}\right)}$$

Fusion Weight Optimization: The fusion coefficient ω was optimized by searching the grid in the following discrete set to determine the value that yields the best overall classification performance.

ω∈{0.00,0.05,0.10,…,1.00}.

For each candidate value, logits were fused according to Equation (3) and independently evaluated in five cross-validation folds. Model selection was based on the mean weighted F1-score computed over folds.

Algorithm 1 summarizes the complete adaptive logit fusion and grid search procedure.
**Algorithm 1** Adaptive logit fusion with grid search1:Define candidate weights Ω={0.00,0.05,…,1.00}2:**for** each ω∈Ω **do**3:    **for** each fold k=1…5 **do**4:             Load trained EfficientNetV2-S and ResNet50V2 models5:             **for** each test batch **do**6:                   Compute logits $z^{\left(E\right)}$ and $z^{\left(R\right)}$7:                   Compute fused logits using Equation (3)8:             **end for**9:             Compute accuracy and weighted F1-score10:  **end for**11:  Compute mean weighted F1-score across folds12:**end for**13:Select $\omega^{*}=\arg\max_{\omega }\mathrm{MeanWeightedF}1$

Overall accuracy is computed over aggregated predictions across all classes and is defined as follows:

(6) $$\mathrm{Accuracy}=\frac{\sum_{i=1}^{C}TP_{i}}{N},$$

where $TP_{i}$ denotes the number of true positive predictions for class *i*, *C* is the total number of classes, and *N* represents the total number of evaluated samples. Accuracy is therefore distinct from macro- and weighted-F1 scores. In single-label multi-class classification, weighted recall is mathematically equivalent to overall accuracy, which explains their identical numerical values in the reported tables. In contrast, the weighted F1-score is computed as the support-weighted average of per-class F1-scores, where each class F1 is the harmonic mean of its precision and recall. Therefore, accuracy and weighted F1-score do not represent the same quantity. In our results, per-class precision and recall are relatively well balanced for the dominant classes, which results in the weighted F1-score numerically close to overall accuracy.

## 3. Experimental Results

This section presents a comprehensive evaluation of the proposed adaptive logit fusion-based ensemble framework for sperm morphology classification. First, the optimal ensemble configuration is identified through a grid search over fusion weights, highlighting the complementary contributions of the two backbone networks. The results of each fold are then aggregated into a cumulative classification report to offer an overall and unbiased assessment of the model performance under class imbalance. Finally, a comparative analysis against individual backbone models and a cumulative confusion matrix is provided to further examine class-wise prediction patterns, error modes, and the effectiveness of the ensemble strategy.

### 3.1. Ensemble Weight Grid Search

Following the optimization protocol described in Section 2.6, the fusion coefficient ω was evaluated over the predefined grid. Table 1 presents fold-wise accuracy and weighted F1-score values for each candidate weight.

For each ω, the mean weighted F1-score across folds was computed to enable direct comparison. The highest mean performance was obtained at ω=0.45, which was therefore selected as the final ensemble configuration.

As shown in Table 1, performance improves steadily from ω=0.00 to intermediate values, demonstrating the complementary contribution of ResNet50V2 to EfficientNetV2-S. Beyond ω=0.45, performance gradually decreases, indicating that excessive reliance on a single backbone reduces the benefit of balanced logit integration.

### 3.2. Systematic Ablation Analysis

A systematic ablation study was conducted to quantify the incremental contribution of data augmentation, class-weighted loss, and logit-level fusion. All configurations were evaluated under an identical five-fold cross-validation protocol. For each backbone architecture, training was performed under four settings: baseline, class-weighted loss only, data augmentation only, and both augmentation and class-weighted loss. The same progression was applied to the logit-level ensemble. For ensemble configurations, logits were fused using a fixed weight of ω=0.50. The final row reports the optimized fusion weight ω=0.45 as determined by grid search.

As shown in Table 2, data augmentation yields the most substantial performance gain for both backbone architectures, providing an absolute F1 improvement of +0.0916 for EfficientNetV2-S and +0.0428 for ResNet50V2 relative to their respective baselines. The incorporation of class-weighted loss further improves performance under class imbalance, although its isolated contribution is comparatively smaller.

Logit-level fusion consistently enhances generalization across all settings. When both augmentation and class weighting are enabled, the ensemble with ω=0.50 improves F1 by +0.0762 over its baseline configuration. The optimized fusion coefficient ω=0.45 yields the highest overall performance, achieving a total improvement of +0.0776 relative to the ensemble baseline. These results confirm that balanced logit integration between complementary backbones provides measurable and consistent gains beyond individual architectures.

### 3.3. In-Depth Classification Report

To provide an overall and unbiased evaluation of the proposed ensemble, predictions obtained from all five folds were aggregated into a single cumulative classification report. This analysis reflects the model’s performance across the entire dataset, capturing both class-level behavior and the impact of class imbalance. Table 3 details the comprehensive classification metrics of the proposed adaptive logit fusion ensemble model across all validation folds, encompassing a total of 18,456 samples. The model demonstrated robust generalization capability, achieving an overall accuracy of 70.94% and a weighted average F1-score of 0.7065. These aggregate metrics indicate that the ensemble strategy effectively handles the multi-class nature of the sperm morphology dataset, maintaining a balanced trade-off between precision (0.7057) and recall (0.7094) on a weighted basis.

**Figure 6:**
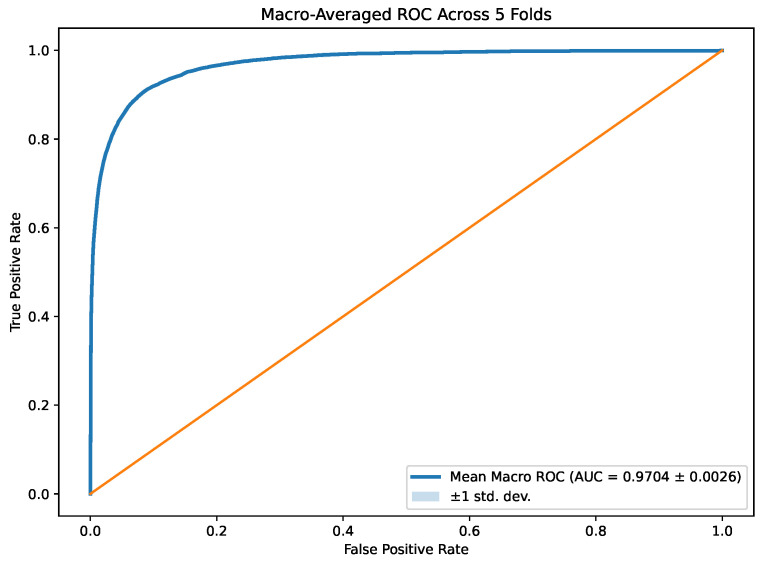
Macro-averaged ROC curve across five folds. The blue solid curve represents the mean ROC, the light blue shaded region indicates ±1 standard deviation (very small and therefore nearly overlapping with the mean curve), and the orange diagonal line denotes the random-classifier baseline.

Figure 6 presents the macro-averaged ROC (receiver operating characteristic) curve computed across five cross-validation folds. The ensemble achieved a mean macro-AUROC of 0.9704 ± 0.0026, indicating strong discriminative performance across the 18 morphology classes. The steep rise of the curve at low false positive rates suggests that the model maintains high sensitivity while limiting false positives, even under pronounced class imbalance. The relatively narrow ±1 standard deviation band further indicates consistent ranking behavior across different train–test partitions. These findings complement the accuracy and F1-score results reported by demonstrating stable probability-based class separability across folds.

An analysis of individual class performances reveals that the model excels in detecting distinct morphological defects. The ‘PinHead’ class yielded near-perfect results with an F1-score of 0.9910, suggesting that its visual features are highly discriminative for the proposed architecture. Similarly, tail defects such as ‘CurlyTail’ and ‘ShortTail’ demonstrated high reliability, with F1-scores of 0.8975 and 0.8717, respectively. Crucially, the ‘Normal’ sperm morphology, which is clinically significant for fertility assessment, was classified with high efficacy (F1-score: 0.8506), supported by a high recall of 0.8763.

The impact of class imbalance is evident, but is notably managed in certain categories. For instance, the ‘DoubleHead’ class, despite having a very low support of 48 samples, achieved a commendable F1-score of 0.7609, indicating that the ensemble model can effectively learn rare but visually distinct features. Conversely, classes with subtle structural deformations presented greater challenges. Specifically, neck anomalies such as ‘AsymmetricNeck’ and ‘ThinNeck’ recorded the lowest performance metrics (F1-scores of 0.2906 and 0.3481, respectively). The low recall rates for these classes (0.2322 and 0.3073) imply that these specific deformities share overlapping feature representations with other classes, leading to a higher rate of false negatives.

Furthermore, a trade-off between precision and recall was observed in prevalent head defects. For the ‘VacuolatedHead’ class, which has substantial support of 1697 samples, the model prioritized sensitivity, achieving a recall of 0.7077 compared to a precision of 0.6295. This indicates that while the model is proficient at identifying vacuolated heads, it tends to generate some false positives. Overall, the results suggest that while the proposed ensemble method provides state-of-the-art performance for distinct shapes and majority classes, subtle neck deformities and extremely rare classes like ‘LongTail’ (F1-score: 0.4857) remain areas for future improvement in feature representation.

The comparative performance of individual base models (ResNet50V2 and EfficientNetV2-S) and the proposed adaptive logit fusion ensemble model is presented in Table 4. The experimental results demonstrate that the proposed ensemble approach yields a superior generalization capability compared to the single backbone architectures. Specifically, the ensemble model achieved the highest overall performance with a weighted average F1-score of 0.7065 and a macro average F1-score of 0.6860, surpassing both ResNet50V2 (0.6857) and EfficientNetV2-S (0.6772). This improvement confirms that fusing the logits of different architectures effectively combines their feature extraction strengths, resulting in more robust classification decisions.

In terms of class-wise analysis, the proposed ensemble method secured the highest F1-scores in 17 out of the 18 morphological classes. Significant performance gains were observed, particularly in classes that are traditionally difficult to distinguish. For instance, in the ‘DoubleHead’ category, the ensemble model reached an F1-score of 0.7609, providing a substantial improvement over ResNet50V2 (0.6714) and EfficientNetV2-S (0.5906). Similarly, for the ’RoundHead’ class, the ensemble outperformed the individual models by achieving a score of 0.5156. Furthermore, in high-performing classes such as ‘PinHead’ and ‘CurlyTail’, the fusion strategy maintained high precision, achieving scores of 0.9910 and 0.8975, respectively.

Although the ensemble model dominated the majority of the categories, distinct behaviors were observed in specific classes due to the variance between base learners. For the ‘LongTail’ class, the ensemble model achieved a marginal advantage (0.4857) over ResNet50V2 (0.4776). Regarding the ‘ThinNeck’ class, where there was a notable discrepancy between the base models (0.3009 for ResNet50V2 vs. 0.3638 for EfficientNetV2-S), the ensemble provided a balanced output (0.3481), compensating for the weaker model’s performance. Despite these minor exceptions, the consistent improvements across the vast majority of classes validate the effectiveness of the proposed adaptive logit fusion strategy in automated sperm morphology analysis.

### 3.4. Cross-Validation Variability

To quantify the stability of the proposed framework under different train–test partitions, variability across the five cross-validation folds was analyzed. Let K=5 denote the number of folds, and let $m_{k}$ represent a performance metric obtained from fold *k*, where k∈{1,…,K}.

The cross-validation mean μ is computed as shown in Equation (7):

(7) $$\mu =\frac{1}{K}\sum_{k=1}^{K}m_{k}$$

The corresponding unbiased standard deviation σ across folds is calculated as shown in Equation (8):

(8) $$\sigma =\sqrt{\frac{1}{K-1}\sum_{k=1}^{K}{\left(m_{k}-\mu \right)}^{2}}$$

In addition to reporting the mean standard deviation ±, 95% confidence intervals (CI) were calculated using the Student-*t* distribution as follows:

(9) $${\mathrm{CI}}_{95\%}=\mu \pm t_{0.975,K-1}\frac{\sigma }{\sqrt{K}},$$

where $t_{0.975,4}=2.776$ for K=5 folds.

This formulation captures variability induced by data partitioning and reflects model robustness under class imbalance and fold-level distribution shifts.

The ensemble model achieved an accuracy of 0.7094±0.0037 (95% CI: [0.7048,0.7140]), a weighted F1-score of 0.7064±0.0037, and a macro F1-score of 0.6845±0.0095 as presented in Table 5. The macro- and weighted-averaged specificity values were 0.9819 and 0.9654, respectively, reflecting strong true negative performance across morphology classes.

For probability-based evaluation, both macro- and micro-averaged discrimination metrics were analyzed. The macro-AUROC and weighted-AUROC computed over aggregated predictions were 0.9700 and 0.9571, respectively. The corresponding macro-AUPRC and weighted-AUPRC values were 0.7214 and 0.7601. In addition, fold-wise micro-averaged metrics were computed to quantify variability across partitions. The micro-AUROC achieved 0.9798±0.0008, while the micro-AUPRC reached 0.7926±0.0035. The low standard deviation of the AUROC indicates highly stable ranking performance across different train–test splits.

All folds were trained independently using identical hyperparameter settings and a fixed random seed. Therefore, the reported variability reflects differences arising from data partitioning rather than repeated stochastic initialization. While additional random restarts per fold could further quantify initialization variance, fold-level variability already provides a reliable estimate of model stability under distributional shifts in this moderately sized and class-imbalanced dataset.

To further evaluate whether the observed improvements are statistically significant across folds, paired statistical tests were conducted on the weighted F1-scores as given in Table 6.

### 3.5. Confusion Matrix Analysis

**Figure 7:**
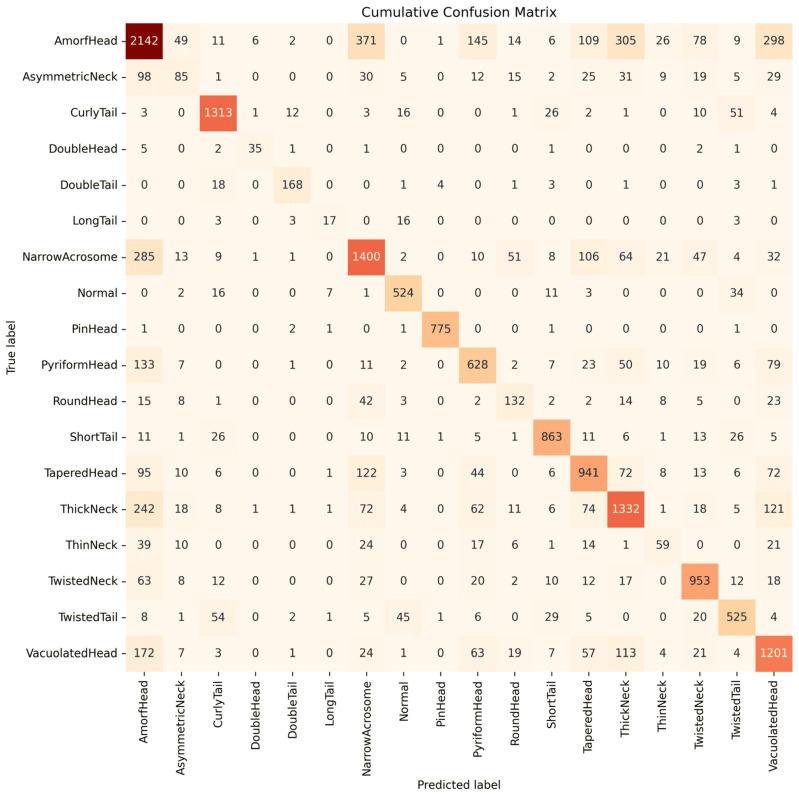
Cumulative confusion matrix aggregated over all five folds for the proposed adaptive logit-fusion ensemble. Rows correspond to ground-truth classes and columns to predicted classes. Diagonal entries represent correct classifications, while off-diagonal values highlight dominant misclassification patterns. Darker color intensity indicates higher sample counts, whereas lighter colors correspond to fewer samples.

To further analyze class-wise prediction behavior and error patterns, a general confusion matrix was constructed by aggregating predictions from all five folds. This representation provides a comprehensive view of how different sperm morphology classes are recognized and confused by the proposed ensemble model, particularly in the presence of class imbalance and subtle inter-class similarities. Figure 7 visualizes the resulting confusion matrix.

The confusion matrix indicates that most misclassifications occur between morphologically similar classes with overlapping visual characteristics. Head-related abnormalities (AmorfHead, PyriformHead, and TaperedHead) show substantial mutual confusion due to ambiguous global shape boundaries and variations in acrosomal appearance, while neck-related classes (ThinNeck and AsymmetricNeck) are frequently misclassified as head or acrosomal abnormalities, reflecting the difficulty of capturing subtle local deformations using appearance-based features alone. Table 7 summarizes these dominant error patterns quantitatively, showing that AmorfHead samples are most often confused with NarrowAcrosome, ThickNeck, and VacuolatedHead, and that PyriformHead and ThinNeck similarly exhibit confusion with morphologically related categories. In contrast, visually distinctive classes such as PinHead and DoubleTail demonstrate strong diagonal dominance with minimal confusion, highlighting the effectiveness of the proposed model in recognizing pronounced structural abnormalities.

### 3.6. External Validation on HuSHeM

To evaluate cross-dataset generalization under dataset shift, the proposed adaptive logit fusion framework was additionally validated on the Human Sperm Head Morphology (HuSHeM) dataset introduced by Shaker et al. [26]. HuSHeM is a publicly available benchmark dataset designed for sperm head morphology classification and includes four expert-annotated classes corresponding to Normal, Pyriform, Tapered, and Amorphous sperm heads. The final dataset consists of 216 RGB sperm head images with a resolution of 131×131 pixels.

HuSHeM represents an independent acquisition domain with imaging conditions and annotation distributions that differ from Hi-LabSpermMorpho. While Hi-LabSpermMorpho covers head, neck, and tail abnormalities across 18 classes, HuSHeM focuses exclusively on head morphology, providing a complementary external benchmark for assessing the robustness and generalization capability of the proposed framework. The same five-fold cross-validation protocol and training configuration were preserved, with only input resizing adapted to account for differences in image resolution. All folds maintained identical total sample counts and class distributions.

Across the five folds, the proposed method achieved a mean accuracy of 95.04±5.26% and a mean weighted F1-score of 0.950±0.052. The aggregated overall performance across all folds reached 94.91% accuracy and a weighted F1-score of 0.9492, demonstrating consistent classification behavior on an external dataset. The optimal ensemble weight ω varied across folds (0.0–0.7), indicating that the complementary contributions of EfficientNetV2-S and ResNet50V2 remain dataset dependent while overall performance stability is preserved.

### 3.7. Computational Complexity and System Configuration

All experiments were conducted on Ubuntu 24.04 LTS running on a workstation equipped with an AMD Ryzen 5 5600X CPU (central processing unit) and an NVIDIA GeForce RTX 5070 Ti GPU with 16 GB VRAM (video random access memory). The implementation was developed using PyTorch 2.9.0 with CUDA (Compute Unified Device Architecture) 13.0 and cuDNN (CUDA Deep Neural Network library) 9.13. The software stack additionally included torchvision 0.24.0, NumPy 2.3.5, scikit-learn 1.7.2, and Albumentations 2.0.8.

Training and evaluation were performed with a fixed random seed (42) applied to Python, NumPy, and PyTorch to ensure reproducibility. cuDNN deterministic mode was enabled, and each fold in the five-fold cross-validation protocol was trained independently under identical hyperparameter settings. Data loading employed deterministic shuffling with fixed seed control and mixed-precision training (AMP). Inference benchmarks were performed on the GPU using a batch size of 1 at an input resolution of 240×240.

Model complexity was quantified in terms of parameter count and theoretical floating point operations (FLOPs), computed using fvcore under identical input dimensions. Inference latency was measured over 200 iterations after 100 warm-up runs using synchronized CUDA timing. EfficientNetV2-S contains 20.20 M parameters with 3.39 GFLOPs (giga floating point operations), while ResNet50V2 contains 23.54 M parameters with 4.95 GFLOPs. The proposed adaptive logit fusion ensemble combines both backbones, resulting in 43.74 M parameters and 8.34 GFLOPs.

In terms of inference speed as measured in frames per second (FPS), EfficientNetV2-S achieved a median latency of 10.82 ms per image (92.43 FPS), whereas ResNet50V2 achieved 6.54 ms per image (152.99 FPS). The ensemble model required 16.96 ms per image (58.95 FPS). These results indicate that the proposed ensemble improves classification accuracy with a moderate increase in computational cost while maintaining real-time feasibility on modern GPU hardware.

## 4. Discussion

This section presents a comprehensive interpretation of the experimental findings in relation to existing studies. A comparative analysis with prior work is provided, followed by an examination of class-wise performance characteristics and cumulative trends observed across cross-validation folds. The influence of architectural design and training strategies is further analyzed to clarify their contribution to the reported improvements. Particular attention is given to the impact of morphological distinctiveness and inter-class similarity on classification behavior, as well as to the role of ensemble learning and efficient optimization techniques in enhancing robustness and generalization.

### 4.1. Comparison with Related Work

Table 8 summarizes representative studies that employed the BesLab subset of the Hi-LabSpermMorpho dataset, highlighting differences in methodological design and evaluation protocols. The baseline study by Aktaş et al. [8] benchmarked individual CNN and Transformer architectures under a five-fold cross-validation protocol and reported notable confusion among visually similar classes. Later works extended this baseline by introducing alternative ensemble formulations, including multi-level feature fusion [27] and category-aware two-stage frameworks [28], achieving incremental performance improvements under the same dataset subset and five-fold evaluation setting.

All compared studies report results on the BesLab subset evaluated using five-fold cross-validation. The present study follows the official fold configuration. Performance values of prior works are taken directly from their respective publications. While minor differences in preprocessing, input resolution, or optimization schedules may exist across studies, all results correspond to the same BesLab subset under five-fold cross-validation, enabling dataset-level comparability.

In contrast to prior ensemble-based studies that combine intermediate feature representations or rely on multi-stage fusion pipelines [27,29], the present work adopts a late fusion strategy operating directly in pre-softmax logit space. This formulation avoids feature-level aggregation and additional classifier modules, preserving independently learned decision boundaries of each backbone network while enabling adaptive balancing through a single weighting parameter.

Compared with the multi-level ensemble method in [27], which integrates information at the feature representation level, the proposed method performs fusion directly in logit space, enabling post-training combination of model outputs after independent learning and preserving class-level decision information prior to softmax normalization. Similarly, the category-aware divide-and-ensemble framework proposed in [28] introduces a two-stage formulation where samples are first grouped into coarse morphology categories before specialized classification. In contrast, the proposed framework applies weighted logit integration only at the final prediction stage using parallel backbone models.

Furthermore, it is important to explicitly distinguish the proposed approach from other related studies in the broader literature. While the foundational study by Aktaş et al. [8] established the dataset baseline by evaluating individual single-stage architectures, our work extends this by demonstrating the superior generalization of adaptive logit-level fusion using complementary parallel backbones. Unlike the work of Shahzad et al. [20], which utilized sequential deep neural networks for sperm abnormality detection, our framework leverages parallel heterogeneous backbones (EfficientNetV2-S and ResNet50V2) to capture spatial features simultaneously. Finally, while Hellín et al. [16] provided a comprehensive analysis of the impact of class imbalance on deep learning models in general medical imaging, our study actively mitigates this issue by integrating a class-weighted cross-entropy loss directly with the logit fusion strategy, offering a tailored solution for the severe imbalance inherent in fine-grained sperm morphology classification.

Overall, the main distinction of this study lies in demonstrating that adaptive logit-level fusion of complementary CNN backbones, evaluated under a consistent five-fold cross-validation protocol, can provide improved performance while maintaining a unified single-stage prediction formulation.

### 4.2. Overall Performance

The aggregated results across all five folds demonstrate an overall classification accuracy of 70.94% and a macro-averaged F1-score of 68.60%. These findings indicate that the use of a class-weighted loss function effectively mitigates bias toward overrepresented classes, leading to more balanced decision boundaries across the full set of morphological categories.

While the overall performance confirms the suitability of deep CNN-based models for sperm morphology classification, certain morphological ambiguities remain challenging. In particular, overlaps between visually similar classes—such as LongTail and TwistedTail, or AmorfHead and other head-related abnormalities—continue to result in misclassifications, suggesting inherent limitations of appearance-based feature representations in fine-grained morphology discrimination.

Although standalone backbone models—particularly ResNet50V2—already achieved strong performance, the proposed adaptive logit fusion-based ensemble consistently improved precision and recall in multiple folds. This gain indicates that the two architectures capture complementary feature representations, allowing the ensemble to compensate for individual model weaknesses.

EfficientNetV2-S demonstrated favorable accuracy-to-parameter efficiency through its compound scaling strategy and optimized MBConv blocks, benefiting from large-scale pretraining on ImageNet-21k and ImageNet-1k. In contrast, ResNet50V2’s deep residual structure and pre-activation design, combined with knowledge distillation, facilitated stable gradient propagation and robust feature learning. The fusion of these complementary architectural properties resulted in a more reliable and consistent classification behavior.

In addition, the use of automatic mixed precision (AMP) training with dynamic gradient scaling significantly reduced memory consumption and accelerated training without degrading model accuracy. For both backbones, training was performed with and without AMP under the same configuration. For EfficientNetV2-S, AMP reduced GPU memory usage by 46.6% and achieved a 70.3% training speedup, whereas for ResNet50V2, the memory footprint remained within the same range while still providing a 17.4% speedup. Test accuracy and weighted F1-score differed by less than 0.1% between FP32 and AMP configurations. Overall, the combination of ensemble learning, class-aware optimization, and efficient training strategies contributed to a smoother decision process and improved generalization performance across diverse sperm morphology classes without introducing additional architectural complexity.

### 4.3. Limitations

Despite the strong performance achieved by the proposed adaptive logit-level ensemble framework, several limitations should be acknowledged.

A primary limitation concerns severe class imbalance. Although class-weighted loss and augmentation improved minority class recognition, extremely rare categories such as LongTail continue to exhibit lower F1-scores compared to visually distinctive abnormalities. This indicates that loss reweighting alone cannot fully compensate for the limited representation of rare morphological patterns. In addition to class-weighted optimization and conventional augmentation, generative data balancing approaches such as CycleGAN-based image-to-image translation could be explored to increase minority class representation. However, generative adversarial network (GAN)-based methods typically require large-scale training data for stable learning, and were therefore not considered within the scope of this study. While beyond the scope of the present study, such methods may provide complementary strategies for addressing severe imbalance in medical morphology datasets.

Another limitation relates to the exclusive reliance on appearance-based convolutional feature extraction without explicit structural modeling. Subtle neck abnormalities, including ThinNeck and AsymmetricNeck, remain challenging due to overlapping visual characteristics. The absence of segmentation-guided or shape-aware representations may restrict the model’s ability to capture fine-grained geometric differences.

The ensemble strategy also increases computational complexity compared to individual backbone models. Although real-time inference remains feasible on modern GPU hardware, the combined parameter count and floating-point operations approximately double relative to a single architecture. This trade-off between accuracy and efficiency may influence deployment decisions in practical clinical environments.

Addressing these limitations requires improved imbalance-aware learning strategies, enhanced structural modeling mechanisms, and efficiency-oriented model design.

## 5. Conclusions

This study introduced a logit-level fusion framework combining EfficientNetV2-S and ResNet50V2 for 18-class sperm morphology classification under severe class imbalance. Using five-fold cross-validation on the BesLab subset of Hi-LabSpermMorpho, the proposed ensemble achieved an overall accuracy of 70.94%, a weighted F1-score of 70.65%, and a macro F1-score of 68.60%. The model demonstrated strong discriminative capability with a macro-AUROC of 0.9704 and stable fold-level variability. Statistical analysis confirmed that performance improvements over individual backbones were significant. The ablation study further established that data augmentation and logit-level fusion were the primary contributors to performance gains, while class-weighted optimization provided additional improvement under imbalance. External validation on the HuSHeM dataset yielded 94.91% accuracy, confirming robustness under dataset shift.

Future research may explore segmentation-guided modeling to better preserve morphological structure, imbalance-aware generative augmentation for rare classes, and evaluation across multi-center datasets with diverse acquisition protocols. In addition, hybrid CNN–Transformer architectures and uncertainty-aware inference mechanisms represent promising directions for improving fine-grained discrimination. These extensions could enhance reliability in clinically sensitive morphology categories and support the development of clinically deployable decision-support systems for automated fertility assessment.

## Figures and Tables

**Table 1:** Grid search results for logit fusion weight ω combining EfficientNetV2-S and ResNet50V2 logits. Bold values denote the highest accuracy or F1 score within each fold among all evaluated fusion weights.

Weight (ω)	Fold 1		Fold 2		Fold 3		Fold 4		Fold 5	
	Acc	F1	Acc	F1	Acc	F1	Acc	F1	Acc	F1
0.0000	0.6848	0.6833	0.6822	0.6800	0.6891	0.6868	0.6913	0.6890	0.6930	0.6899
0.0500	0.6869	0.6855	0.6868	0.6846	0.6910	0.6886	0.6924	0.6903	0.6989	0.6960
0.1000	0.6897	0.6880	0.6911	0.6889	0.6951	0.6927	0.6932	0.6910	0.7014	0.6983
0.1500	0.6943	0.6925	0.6951	0.6932	0.6996	0.6953	0.6978	0.6956	0.7036	0.7008
0.2000	0.6992	0.6973	0.6981	0.6959	0.7013	0.6987	0.7005	0.6984	0.7044	0.7013
0.2500	0.7019	0.6997	0.7038	0.7013	0.7075	0.7009	0.6992	0.6969	0.7079	0.7047
0.3000	0.7005	0.6981	0.7059	0.7035	0.7075	0.7048	0.7022	0.6999	0.7106	0.7078
0.3500	0.7024	0.6999	0.7103	0.7077	0.7091	0.7075	0.7043	0.7019	0.7110	0.7078
0.4000	0.7032	0.7006	0.7141	0.7117	0.7101	0.7064	0.7043	0.7015	0.7117	0.7083
**0.4500**	**0.7049**	**0.7023**	0.7141	0.7117	**0.7100**	**0.7064**	**0.7065**	**0.7035**	**0.7117**	**0.7083**
0.5000	0.7035	0.7007	**0.7162**	**0.7138**	0.7097	0.7062	0.7055	0.7025	0.7111	0.7063
0.5500	0.7046	0.7018	0.7151	0.7128	0.7098	0.7054	0.7057	0.7024	0.7109	0.7073
0.6000	0.7040	0.7013	0.7143	0.7118	0.7089	0.7054	0.7033	0.7001	0.7107	0.7073
0.6500	0.6992	0.6962	0.7149	0.7123	0.7078	0.7043	0.7016	0.6980	0.7072	0.6984
0.7000	0.6970	0.6941	0.7132	0.7105	0.7057	0.7022	0.7012	0.6971	0.6954	0.6915
0.7500	0.6970	0.6941	0.7065	0.7037	0.7035	0.7016	0.7014	0.6971	0.6954	0.6915
0.8000	0.6959	0.6931	0.7057	0.7030	0.6995	0.6925	0.6950	0.6910	0.6913	0.6874
0.8500	0.6910	0.6884	0.7030	0.7005	0.6942	0.6882	0.6967	0.6918	0.6870	0.6829
0.9000	0.6869	0.6844	0.6984	0.6963	0.6864	0.6824	0.6927	0.6881	0.6870	0.6829
0.9500	0.6807	0.6781	0.6922	0.6893	0.6797	0.6762	0.6881	0.6841	0.6824	0.6782
1.0000	0.6769	0.6740	0.6849	0.6834	0.6797	0.6762	0.6827	0.6777	0.6786	0.6744

**Table 2:** Comprehensive ablation study evaluating augmentation, class-weighted loss, and logit fusion under five-fold cross-validation. The Δ column indicates F1-score improvement relative to the baseline of each model. Bold values indicate the best overall performance among all evaluated configurations.

Model Architecture	Augmentation	Class-Weighted Loss	Accuracy	Weighted F1-Score	Δ Weighted F1-Score
EfficientNetV2-S	No	No	0.5745	0.5692	–
EfficientNetV2-S	No	Yes	0.5799	0.5742	+0.0050
EfficientNetV2-S	Yes	No	0.6716	0.6608	+0.0916
EfficientNetV2-S	Yes	Yes	0.6784	0.6772	+0.1080
ResNet50V2	No	No	0.6366	0.6319	–
ResNet50V2	No	Yes	0.6380	0.6372	+0.0053
ResNet50V2	Yes	No	0.6827	0.6747	+0.0428
ResNet50V2	Yes	Yes	0.6930	0.6857	+0.0538
Logit Fusion (ω=0.50)	No	No	0.6347	0.6289	–
Logit Fusion (ω=0.50)	No	Yes	0.6513	0.6444	+0.0155
Logit Fusion (ω=0.50)	Yes	No	0.7002	0.7010	+0.0721
Logit Fusion (ω=0.50)	Yes	Yes	0.7082	0.7051	+0.0762
Logit Fusion (ω=0.45)	Yes	Yes	**0.7094**	**0.7064**	**+0.0776**

**Table 3:** Comprehensive classification results of the proposed adaptive logit fusion-based ensemble approach (ω=0.45) across all folds.

Class	Precision	Recall	F1-Score	Support
AmorfHead	0.6467	0.5997	0.6223	3572
AsymmetricNeck	0.3881	0.2322	0.2906	366
CurlyTail	0.8854	0.9099	0.8975	1443
DoubleHead	0.7955	0.7292	0.7609	48
DoubleTail	0.8660	0.8400	0.8528	200
LongTail	0.6071	0.4048	0.4857	42
NarrowAcrosome	0.6531	0.6811	0.6668	2054
Normal	0.8265	0.8763	0.8506	598
PinHead	0.9910	0.9910	0.9910	782
PyriformHead	0.6193	0.6421	0.6305	978
RoundHead	0.5176	0.5136	0.5156	257
ShortTail	0.8726	0.8708	0.8717	991
TaperedHead	0.6799	0.6726	0.6762	1399
ThickNeck	0.6633	0.6737	0.6685	1977
ThinNeck	0.4014	0.3073	0.3481	192
TwistedNeck	0.7824	0.8258	0.8035	1154
TwistedTail	0.7554	0.7436	0.7495	706
VacuolatedHead	0.6295	0.7077	0.6663	1697
**Accuracy**			0.7094	18,456
**Macro Avg**	0.6989	0.6790	0.6860	18,456
**Weighted Avg**	0.7057	0.7094	0.7065	18,456

**Table 4:** Comparison of the per-class F1-scores of ResNet50V2, EfficientNetV2-S, and the proposed adaptive logit fusion-based ensemble model. Bold values indicate the best F1-score among the compared models for each class.

Class	ResNet50V2	EfficientNetV2-S	Adaptive Logit Fusion Ensemble
AmorfHead	0.6058	0.5796	**0.6223**
AsymmetricNeck	0.2861	0.2116	**0.2906**
CurlyTail	0.8931	0.8714	**0.8975**
DoubleHead	0.6714	0.5906	**0.7609**
DoubleTail	0.8388	0.7760	**0.8528**
LongTail	0.4776	0.4600	**0.4857**
NarrowAcrosome	0.6607	0.6186	**0.6668**
Normal	0.8348	0.8195	**0.8506**
PinHead	0.9878	0.9853	**0.9910**
PyriformHead	0.6015	0.5992	**0.6305**
RoundHead	0.4694	0.4699	**0.5156**
ShortTail	0.8543	0.8480	**0.8717**
TaperedHead	0.6541	0.6402	**0.6762**
ThickNeck	0.6569	0.6383	**0.6685**
ThinNeck	0.3009	**0.3638**	0.3481
TwistedNeck	0.7864	0.7790	**0.8035**
TwistedTail	0.7163	0.7104	**0.7495**
VacuolatedHead	0.6424	0.6495	**0.6663**
**Macro Avg**	0.6653	0.6430	**0.6860**
**Weighted Avg**	0.6857	0.6772	**0.7065**

**Table 5:** Cross-validation performance reported as mean ± standard deviation across five folds.

Metric	Mean ± Std
Accuracy	0.7094±0.0037
Macro Precision	0.6996±0.0147
Macro Recall	0.6786±0.0092
Macro F1-score	0.6845±0.0095
Weighted Precision	0.7068±0.0029
Weighted Recall	0.7094±0.0037
Weighted F1-score	0.7064±0.0037

**Table 6:** Paired statistical significance analysis of weighted F1-score across five folds.

Comparison	Mean Diff.	*t*-Value	*p*-Value	Significant (α=0.05)
Ensemble vs. ResNet50V2	0.0208	7.01	0.0022	Yes
Ensemble vs. EfficientNetV2-S	0.0294	24.43	$1.7\times 10^{-5}$	Yes

**Table 7:** Most frequently confused sperm morphology classes.

True Class	Most Confused with	Misclassified
AmorfHead	NarrowAcrosome, ThickNeck, VacuolatedHead	371, 305, 298
ThinNeck	AmorfHead, NarrowAcrosome, VacuolatedHead	39, 24, 21
PyriformHead	AmorfHead, VacuolatedHead, ThickNeck	133, 79, 50

**Table 8:** Comparison of representative studies using the Hi-LabSpermMorpho dataset, restricted to the BesLab subset (18,456 images) for direct comparability with this work. The table contrasts the main methodological ideas, evaluation protocols, and reported performance characteristics of each approach.

Study	Main Idea/Methodology	Evaluation Setting	Reported Results on BesLab
Aktaş et al. (2024) [8]	Comprehensive benchmarking of single-stage CNN and Vision Transformer architectures to establish baseline performance across diverse model families	Stain-specific evaluation using fixed training and test partitions	EfficientNetV2-M obtained the highest accuracy among the evaluated models on BesLab (65.05%), with notable confusion between morphologically similar classes
Aktaş et al. (2025) [27]	Multi-level ensemble framework combining features from multiple EfficientNetV2 variants with classical machine learning classifiers through soft voting	BesLab-only evaluation with a predefined train–test split	The ensemble configuration increased BesLab accuracy to 67.70% relative to single-backbone baselines
Türkoğlu et al. (2025) [28]	Category-aware two-stage divide-and-ensemble strategy that first separates samples into coarse morphological groups before applying specialized CNN and Transformer models	Stain-wise evaluation with hierarchical routing	The proposed framework reported an accuracy of 69.43% on the BesLab subset
**This study**	Adaptive logit-level fusion of EfficientNetV2-S and ResNet50V2 backbones, focusing on complementary representations without additional hierarchical decision stages	BesLab-only evaluation using five-fold cross-validation	An overall accuracy of 70.94% was obtained, together with macro-F1 (68.60%) and weighted-F1 (70.65%) metrics

## Data Availability

The datasets utilized in this work, namely Hi-LabSpermMorpho, can be found in online repositories. Repository names and accession numbers: https://github.com/Yildiz-Hi-Lab/Hi-LabSpermMorpho (accessed on 16 December 2025).

## Abbreviations

The following abbreviations are used in this manuscript:

AMP	Automatic Mixed Precision
AUPRC	Area Under the Precision-Recall Curve
AUROC	Area Under the Receiver Operating Characteristic Curve
BiT	Big Transfer
CI	Confidence Interval
CNN	Convolutional Neural Network
CPU	Central Processing Unit
CUDA	Compute Unified Device Architecture
cuDNN	CUDA Deep Neural Network library
FLOPs	Floating Point Operations
FPS	Frames Per Second
GAN	Generative Adversarial Network
GFLOPs	Giga Floating Point Operations
GPU	Graphics Processing Unit
HuSHeM	Human Sperm Head Morphology
MBConv	Mobile Inverted Bottleneck Convolution
RGB	Red, Green, Blue
ROC	Receiver Operating Characteristic
VRAM	Video Random Access Memory
WHO	World Health Organization
